# Whispering-gallery microlasers for cell tagging and barcoding: the prospects for in vivo biosensing

**DOI:** 10.1038/s41377-021-00517-6

**Published:** 2021-04-14

**Authors:** Nikita Toropov, Frank Vollmer

**Affiliations:** Department of Physics and Astronomy, Living Systems Institute, University of Exeter, Exeter, EX4 4QD UK

**Keywords:** Imaging and sensing, Biophotonics

## Abstract

Researchers in the field of whispering-gallery-mode (WGM) microresonators have proposed biointegrated low-threshold WGM lasers, to enable large-scale parallel single-cell tracking and barcoding. Although the reported devices have so far been primarily investigated in model applications, most recent results represent important steps towards the development of in vivo tags and sensors that utilize the unique and narrow spectral features of miniature WGM lasers.

## Abstract (web summary)

Omnidirectional lasing particles in living cells and line-by-line sweeping have revealed new prospects for cell tagging and tracking.

## Graphical abstract

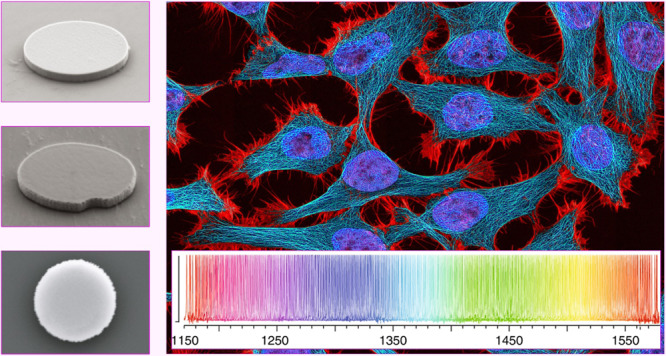

The past 10 years have witnessed the first demonstrations of lasing from within live cells^1^, and based on the phenomenon of whispering-gallery waves or modes (WGMs)^2,3^. Unlike many conventional lasers, WGM lasers can be miniaturized to a size of just a few micrometers that fit inside single cells. They emit coherent light under optical excitation, with lasing thresholds down to 0.13 of picojoules^4^, using excitation power levels that are typical in microscopy and compatible with cells^5^. Confining the light on WGM by near-total internal reflection, results in prolonged photon lifetimes, and high Q-factors, that combined with the small WGM mode volume *V*, are key for achieving low-threshold lasing. A recent paper by Seok-Hyun Yun and Yunfeng Xiao et al.^6^ builds on the unique properties of WGM microlaser, by developing tiny 2 µm by 200-nm semiconductor laser disks with unique and narrow (<0.3 nm) spectral signatures and omnidirectional light emission, for tracking cells over a prolonged time on a confocal microscope. The stage is set, for developing exciting applications in biology, health, and environment using the WGM microcavities as a platform technology that can combine in vivo sensing and cell tracking with state-of-the-art detection capabilities down to single molecules^7^.

Without gain media, passive WGM microresonators have been used over the past 20 years to demonstrate remarkable sensing capabilities, such as single-virus detection by monitoring the WGM frequency shifts with a MHz precision as the nanoparticles and molecules bind to the cavity^8,9^. Further boosting the WGM frequency shift signal, by increasing the intensity of the probing light field at the surface of the cavities with plasmonic nanoparticles, led to demonstrations of single-molecule sensing, detection of single-atomic ions^10^, monitoring of attomolar chemical reactions^11^, and characterizing the conformational changes and characterizing the conformational changes of single enzymes^12^.

Fluorescence-based techniques, on the other hand, have a long history in single-molecule detection, e.g., see review^13^. They have been predominantly used in biology, for biomedical investigations in imaging, sensing, and also high-resolution cell tracking^14–16^. The fluorescence light emission is highly specific, and several channels can be combined in multiplexed experiments^17^. FRET in a Fabry–Perot resonator enabled individual molecule sensitivity for dopamine, nicotine, and single-strand DNA detection^18^. Most recently, single-molecule FRET and live-cell imaging of metabotropic glutamate receptor 2 (mGluR2), a G protein-coupled receptor, showed interconverts between four conformational states, two of which were previously unknown^19^.

Combining the WGM microcavities with fluorophores and other types of gain media provides an exciting new perspective for biosensing^5^. Especially in the important and emerging area of in vivo biosensing, the WGM microlasers enable new ways for probing cells, for taking measurements inside cells and organisms, and for further improving the detection limit and expanding the sensing modalities of the passive WGM sensors. The miniature size, biocompatible materials, and the low WGM lasing thresholds make it possible to integrate the WGM lasers with live cells, for a prolonged time and without altering important physiological functions of the cell^4,6,20,21^. Interestingly, the WGM microspheres are readily taken up by most cells, in a process known as endocytosis^22^. WGM beads inside cells have already been used for in vitro sensing of biomechanical forces and without the need for any special surface modification or coating of the sensor which, however, in some cases can promote the uptake of the laser particles with diameter up to 20 μm^20^. The endocytosis of WGM beads has been shown with different types of cells including human umbilical vein endothelial cells (HUVECs)^22^, HeLa cells^2,6^, different types of cardiac cells^21^, primary macrophages and astrocytes as well as the cell lines HEK 293, NIH 3T3, N7, and SH-SY5Y^20^.

Using microscopy-based techniques for the excitation and analysis of the WGM lasing spectra is a powerful approach for developing applications in biology. The important application of microscopy-based cell tagging and tracking can be automated, for investigating organ development, morphogenesis, spreading of cancer cells, etc.^23–25^. Some of the biology applications will require the labeling of many (potentially thousands of) cells in parallel, and that each cell can be followed over prolonged time intervals from hours to days by measuring a unique spectral signature. Here, WGM lasers can outperform the fluorophore-based cell labeling and tagging approaches. WGM lasers provide unique narrow-linewidth lasing spectra which allows the barcoding of thousands of cells, each hosting one or more of a WGM laser with unique emission wavelength(s) (Fig. 1a^26^). This promising approach to cell barcoding and tagging, however, suffers one important drawback. When using WGM microlasers fabricated from planar semiconductor materials such as InAlGaAs and InGaAsP wafers, which are particularly suitable for developing these applications^25^, the microdisk lasers provide laser emission only in the in-plane direction of the disk, with no appreciable amount of emission expected in the out of plane direction for the transverse electric (TE) modes of the disk. This leads to large fluctuations and loss of the micro-disk laser tracking signals as the microdisks adopt various different orientations inside the cells over time.

**Fig. 1:**
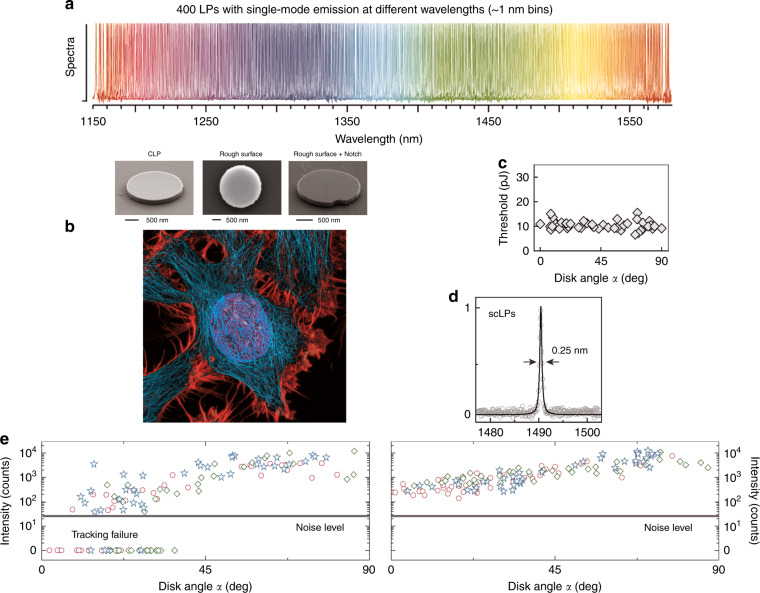
Whispering-gallery microlasers for cell tagging. **a** 400 laser particles with single-mode emission at different wavelengths. **b** HeLa cell and semiconductor laser particles without defects (CLP) and with defects introduced on the surface giving omnidirectional emission. **c** Lasing intensity threshold versus disk angle α of lasing particles with rough sidewalls. **d** Typical emission spectrum of a scatterer-coated laser particle fitted with a Lorentzian lineshape, showing a full width at half maximum of 0.25â€‰nm. **e** Lasing intensity versus disk angle α: left—conventional laser particles, right—omnidirectional laser particles. Reproduced from National Institutes of Health (creator: Tom Deerinck, NIGMS, NIH) and from refs. ^6,26^.

Now, Tang et al.^6^ have found a compelling solution to this problem. They demonstrated omnidirectional emission from semiconductor InGaAsP microdisk lasers by incorporating light scattering into the cavity (Fig. 1b). For this, two designs for introducing scattering nanostructures have been explored: one introduces a boundary defect (a 200 nm notch or a bump) into the 2 μm disk cavity, and the other uses tiny nanoparticles sparsely distributed over the disk resonators and added via the spin-coating and PECVD techniques.

Despite introducing scattering, single-mode lasing was possible with a low lasing threshold of 10 pJ, a narrow linewidth of 0.25 nm (Fig. 1c, d) and with a minimum-to-maximum ratio of the angle-dependent intensity improving from 0.007 (−24 dB) to >0.23 (−6 dB), and nearly independent of the orientation of the tiny semiconductor microdisk lasers (Fig. 1e).

The reliability of cell tracking with the nearly omnidirectional emission provided by the laser disks was tested with HeLa cells. The transfer of the disk to the inside of the cell body is easy: this is accomplished by simply dispersing the disks in cell culture medium from where they randomly distribute and make contact with the cells. The disks were taken up by a process called micropinocytosis. Using a confocal microscope, lasing emission of the disks from inside the cells is recorded on a spectrometer. For excitation, a pump laser (1060–1070 nm, pulse duration 3 ns, repetition rate 2 MHz) was coupled to a side port of the laser-scanning unit of the microscope. Confocal microscopy requires spatial scanning of the excitation lasers which means that it takes minutes to construct the image, depending on the volume of the scan. The emission from the cells could be tracked continuously for 2 h and reliable observed for particles in the cytoplasm of cells without losing the signals. According to the authors suggestion, to enable long-term operation in an aqueous biological environment, semiconductor lasing particles may need an additional protective layer^25^.

Now that the problem of directionality of microdisk laser tags is solved, further improvements of the technique may focus on achieving more rapid and parallel detection of disk barcodes, in real-world in vivo sensing and tracking applications and to answer biological questions^24^. Furthermore, it would be intriguing to explore if cell tracking and barcoding with WGM microdisk lasers can be combined with sensing, perhaps even down to the single-molecule level, by using the plasmonic nanoparticles as the scattering nanostructures.

## References

[1] Gather MC, Yun SH (2011). Single-cell biological lasers. Nat. Photonics.

[2] Humar M, Yun SH (2015). Intracellular microlasers. Nat. Photonics.

[3] Schubert M (2015). Lasing within live cells containing intracellular optical microresonators for barcode-type cell tagging and tracking. Nano Lett..

[4] Fikouras AH (2018). Non-obstructive intracellular nanolasers. Nat. Commun..

[5] Toropov N (2021). Review of biosensing with whispering-gallery mode lasers. Light Sci. Appl..

[6] Tang SJ (2021). Laser particles with omnidirectional emission for cell tracking. Light Sci. Appl..

[7] Vollmer, F. & Yu, D. *Optical Whispering Gallery Modes for Biosensing* (Springer, 2020).

[8] Shao LB (2013). Detection of single nanoparticles and lentiviruses using microcavity resonance broadening. Adv. Mater..

[9] Vollmer F, Arnold S, Keng D (2008). Single virus detection from the reactive shift of a whispering-gallery mode. Proc. Natl. Acad. Sci. USA.

[10] Baaske MD, Vollmer F (2016). Optical observation of single atomic ions interacting with plasmonic nanorods in aqueous solution. Nat. Photonics.

[11] Vincent S, Subramanian S, Vollmer F (2020). Optoplasmonic characterisation of reversible disulfide interactions at single thiol sites in the attomolar regime. Nat. Commun..

[12] Kim E (2017). Label-free optical detection of single enzyme-reactant reactions and associated conformational changes. Sci. Adv..

[13] Möckl L, Moerner WE (2020). Super-resolution microscopy with single molecules in biology and beyond–essentials, current trends, and future challenges. J. Am. Chem. Soc..

[14] Dai L (2020). Realization of a time-correlated photon counting technique for fluorescence analysis. Biomed. Opt. Express.

[15] Hellenkamp B (2018). Precision and accuracy of single-molecule FRET measurements—a multi-laboratory benchmark study. Nat. Methods.

[16] Halabi EA (2020). Dual-activatable cell tracker for controlled and prolonged single-cell labeling. ACS Chem. Biol..

[17] Maetzig T (2017). Lentiviral fluorescent genetic barcoding for multiplex fate tracking of leukemic cells. Mol. Ther. Methods Clin. Dev..

[18] Cao ZX (2019). Biochemical sensing in graphene-enhanced microfiber resonators with individual molecule sensitivity and selectivity. Light Sci Appl..

[19] Liauw BWH, Afsari HS, Vafabakhsh R (2021). Conformational rearrangement during activation of a metabotropic glutamate receptor. Nat. Chem. Biol..

[20] Schubert M (2017). Lasing in live mitotic and non-phagocytic cells by efficient delivery of microresonators. Sci. Rep..

[21] Schubert M (2020). Monitoring contractility in cardiac tissue with cellular resolution using biointegrated microlasers. Nat. Photonics.

[22] Himmelhaus M, Francois A (2009). In-vitro sensing of biomechanical forces in live cells by a whispering gallery mode biosensor. Biosens. Bioelectron..

[23] Chen YC, Fan XD (2019). Biological lasers for biomedical applications. Adv. Opt. Mater..

[24] Keller PJ (2013). Imaging morphogenesis: technological advances and biological insights. Science.

[25] Martino N (2019). Wavelength-encoded laser particles for massively multiplexed cell tagging. Nat. Photonics.

[26] Kwok SJJ (2019). Multiplexed laser particles for spatially resolved single-cell analysis. Light Sci. Appl..

